# Radiotherapy for Adult Medulloblastoma: Evaluation of Helical Tomotherapy, Volumetric Intensity Modulated Arc Therapy, and Three-Dimensional Conformal Radiotherapy and the Results of Helical Tomotherapy Therapy

**DOI:** 10.1155/2018/9153496

**Published:** 2018-03-21

**Authors:** Sun Zong-wen, Yang Shuang-yan, Du Feng-lei, Cheng Xiao-long, Li Qinglin, Chen Meng-yuan, Hua Yong-hong, Jin Ting, Hu Qiao-ying, Chen Xiao-zhong, Chen Yuan-yuan, Chen Ming

**Affiliations:** ^1^Department of Oncology, Jining No. 1 People's Hospital, Jining 272100, China; ^2^Department of Radiation Oncology, Zhejiang Key Lab of Radiation Oncology, Zhejiang Cancer Hospital, Hangzhou 310022, China; ^3^Pharmacy Department, Zhejiang Cancer Hospital, Hangzhou 310022, China; ^4^Second Clinical Medical College, Zhejiang Chinese Medical University, Hangzhou 310053, China

## Abstract

**Introduction:**

All adult medulloblastoma (AMB) patients should be treated with craniospinal irradiation (CSI) postoperatively. Because of the long irradiation range, multiple radiation fields must be designed for conventional radiotherapy technology. CSI can be completed in only one session with helical tomotherapy (HT). We evaluated the dose of HT, volumetric intensity modulated arc therapy (VMAT), and three-dimensional conformal radiotherapy (3D-CRT) of AMB and the results of 5 cases of AMB treated with HT.

**Methods:**

Complete craniospinal and posterior cranial fossa irradiation with HT, VMAT, and 3D-CRT and dose evaluation were performed. And results of 5 cases of AMB treated with HT were evaluated.

**Results:**

A large volume of tissue was exposed to low dose radiation in the organs at risk (OAR), while a small volume was exposed to high dose radiation with HT. The conformity and uniformity of the targets were good with HT and VMAT, and the volume of targets exposed to high dose with VMAT was larger than that of HT. The uniformity of 3D-CRT was also good, but the dose conformity was poor. The main toxicity was hematologic toxicity, without 4th-degree bone marrow suppression. There was 3rd-degree inhibition in the white blood cells, hemoglobin, and platelets. The three female patients suffered menstrual disorders during the course of radiation. Two female patients with heavy menstruation suffered 3rd-degree anemia inhibition, and 2 patients suffered amenorrhea after radiotherapy. Although menstrual cycle was normal, the third patient was not pregnant.

**Conclusion:**

CSI with HT is convenient for clinical practice, and the side effects are mild. With good conformity and uniformity, VMAT can also be used for selection in CSI. For poor conformity, 3D-CRT should not be the priority selection for CSI. In female patients, the ovaries should be protected.

## 1. Introduction

Medulloblastoma (MB), which can spread through the cerebrospinal fluid, is a malignant primitive neuroectodermal tumor that originates from the posterior cranial fossa (PCF). A total of 80% of medulloblastoma patients are diagnosed when they are younger than 15 years of age (median age, 5 years) [1]. The incidence of adult medulloblastoma (AMB) is approximately 0.5/100000 [2, 3], accounting for 0.4–1% of adult nervous system tumors [4]. Surgery is the first treatment choice for nonmetastatic MB, and all patients should be treated with craniospinal irradiation (CSI) postoperatively. Because of the long irradiation range, multiple radiation fields must be designed for conventional radiotherapy technology. It is difficult to abut adjacent radiation fields. CSI can be completed in only one session with helical tomotherapy (HT) [5–8], which is convenient for clinical practice.

This study retrospectively analyzed the treatment results of 5 cases of AMB treated with HT at Zhejiang Cancer Hospital. After that we completed the volumetric intensity modulated arc therapy (VMAT) and three-dimensional conformal radiotherapy (3D-CRT) plan retrospectively and evaluated the dose of HT, VMAT, and 3D-CRT.

## 2. Methods

### 2.1. General Information

Five AMB patients (18 years of age and older) who were confirmed by pathology and treated with HT from June 2015 to October 2016 at Zhejiang Cancer Center were enrolled in the study. The Chang staging system [9] was used to evaluate the patients. Acute toxicity was evaluated with Common Toxicity Criteria (CTC) V4.0, and the last follow-up time was in January 2018. Patients were immobilized by the head, neck, and shoulder, and they wore body thermoplastic masks. CT-simulation equipment was used (i.e., Philips Brilliance CT or GE Light Speed), and the scanning range was from the top of the head to the ossa sedentarium. The simulation images were transmitted to an Accuray CT Planning Station Hi-Art Version 5.1.0 workstation, RayStation 4.0V, and Pinnacle Version 9.2 workstation.

### 2.2. Target Definition and Prescription Dose

The brain, spinal cord, PCF, and organs at risk (OAR) were contoured on the simulation CT images layer by layer. The brain and spinal cord were PTV1 (planning target volume, PTV), while the PCF was PTV2. The doses for PTV1 and PTV2 were 30.6–36 Gy/17–20 F and 50.4–54 Gy/28–30 F, respectively.

### 2.3. Planning

All five patients adopted HT plan; VMAT (RayStation 4.0V) and 3D-CRT (Pinnacle Version 9.2) plan were retrospectively designed for dose evaluation. VMAT consisted of 4 parts: the superior part (brain and upper cervical spinal cord) adopted double therapeutic arc (182–178 degrees and 178–182 degrees); the remaining 3 parts (the spinal cord was derived from the lower cervical part, the thoracic, the lumbar spine, and sac) adopted single therapeutic arc (182–178 degree). 3D-CRT plan consisted of 3 abutting plans: the superior part consisted of 90-degree radiation beam and 270-degree radiation beam (lower bound at C2-3); the median and inferior parts consisted of 0-degree radiation beam, 130–140-degree radiation beam, and 220–230-degree radiation beam (beam angle was slightly different among individual patients).

### 2.4. Image Registration and Plan Execution

#### 2.4.1. Image Registration

Before each plan was executed, the head, neck, and body were scanned separately with megavoltage computed tomography (MVCT). The MVCT images were compared with the corresponding planning images, and then the “superior inferior,” “left right,” “anterior posterior,” and “rotation” movement values of the treatment bed were determined. The mean values of the above head, neck, and body directions were confirmed.

#### 2.4.2. Planning Execution

After completing the image registration, the treatment bed was moved, and the plan was executed.

### 2.5. Plan Evaluation

The dose uniformity was evaluated using the dose homogeneity index (DHI). DHI = *D*_5%_/*D*_95%_. *D*_5%_ was the irradiation dose that 5% PTV received, and *D*_95%_ was the irradiation dose that 95% PTV received [10]. A DHI value close to 1 suggested better dose uniformity, and dose conformity was evaluated with the conformity index (CI). CI = (*V*_T,Pi_ × *V*_T,Pi_)/(*V*_T_ × *V*_Pi_). *V*_T,Pi_ is the volume of the target covered by the prescription dose. *V*_T_ is the volume of the target, and *V*_Pi_ is the tissue volume, including the target covered by the prescription dose [11]. A CI close to 1 suggested better conformity. As the prescription doses differed among the 5 patients, the irradiation dose of the OAR was expressed by the percent prescription dose of the percent volume.

### 2.6. Statistical Analysis

Statistical analysis was performed with the Statistical Package for Social Sciences (SPSS, Chicago, IL) software package, version 18.0, for Windows. Single factor analysis of variance was performed. And, in this study, a two-tailed *P* value < 0.05 was considered statistically significant.

## 3. Results

### 3.1. General Data

The general characteristics of the patients and radiotherapy-related parameters are showed in Table 1. Patient 1 received CSI after a second operation, and the PTV 2 for him was the tumor bed. There was residual tumor in the posterior fossa and intramedullary metastasis in patient 2, who needed morphine for lumbar pain and abandoned treatment after 7 fractions of radiotherapy. The T stage could not be judged for patient 3, who underwent surgery at another hospital and received 2 cycles of etoposide plus carboplatin chemotherapy after radiotherapy. Patient 5 received 4 cycles of temozolomide plus cisplatin chemotherapy after radiotherapy.

### 3.2. Dosimetry Results

**Figure 1:**
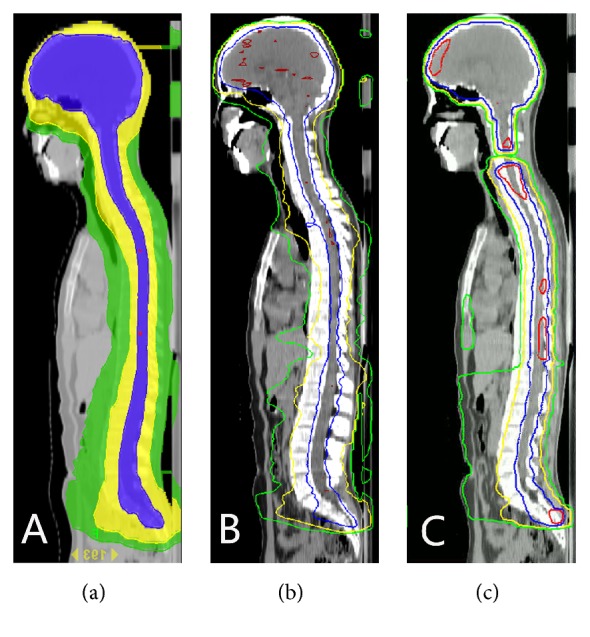
The isodose of (a) HT, (b) VMAT, and (c) 3D-CRT. Red represents 107%, blue 95%, yellow 50%, and green 30%.

HT took the longest CSI beam-on time. VMAT took the second place, and 3D-CRT took the shortest time. The dose uniformity and conformity of HT and VMAT were good. The target volume exposed to high dose (*V*_107%_) in VMAT was larger than that of HT, and 3D-CRT took the highest *V*_107%_. The dose uniformity of 3D-CRT was also good, but the dose conformity was poor. The target dosimetry results were showed in Tables 3 and 4, and the isodose diagram was showed in Figure 1. For HT, *V*_5%_ and *V*_10%_ of the OAR were high, and most *V*_5%_ of OAR was 100%. *V*_20%_ of lens decreased to 0. The remaining *V*_20%_ of OAR decreased rapidly, and *V*_40%_ partially decreased to 0. Most *V*_80%_ of OAR decreased to 0. The dose characteristic of VMAT was similar to that of HT. The dose gradient of HT and VMAT dropped rapidly, while the dose gradient of 3D-CRT dropped slowly. The dosimetry results of OAR were shown in Table 5.

### 3.3. Treatment Toxicity

All patients suffered from headache, dizziness, nausea, and vomiting, which could be relieved with hormones and mannitol. 5-HT3 receptor antagonists were used for patients 1 and 4. Treatment toxicity and clinical treatment are showed in Table 2. The 4 patients who completed the treatment suffered 2nd-degree hair loss after radiotherapy, and their hair eventually returned to normal. During radiotherapy, the main toxicity was hematologic toxicity. There was no 4th-degree bone marrow suppression, but there was a 3rd-degree inhibition of leukocyte and hemoglobin in 2 cases, 3rd-degree platelet inhibition in 1 case, and 2nd-degree neutrophils inhibition in 2 cases. There were menstrual disorders in 3 female patients during the treatment, and 2 patients with heavy menstrual volume suffered 3rd-degree hemoglobin inhibition.

### 3.4. Follow-Up Results

At the time of follow-up, 4 patients had survived, while patient 2 who abandoned radiotherapy died in July 2017. Among the 3 cases of menstrual disorders, the 2 patients who suffered 3rd-degree hemoglobin inhibition had amenorrhea, and the third patient was in a normal menstrual cycle and not pregnant.

## 4. Discussion

The Chang staging was widely used in MB [9, 12, 13]. Craniospinal MRI and CSF examination were helpful in determining whether the tumor was locally residual or intraspinal infiltration, and then the TM stage could be accurately judged [9, 14]. The M stage could be judged only in 2 patients who underwent craniospinal MRI. Residual tumor in the PCF and intraspinal tumor infiltration were showed on the MRI of one patient. Craniospinal MRI and CSF examinations should be completed before CSI; otherwise the tumor stage and patient prognosis cannot be evaluated properly.

Before CSI irradiation, the therapeutic outcomes of MB were poor. In 1953, Paterson and Farr [15] reported using the CSI technique to treat patients with MB, and the 3-year OS was 65%. Then, the CSI technique established its status in the treatment of MB. The target range of CSI is long enough that one radiation field cannot cover the target. Abutting radiation fields is inevitable in two-dimensional radiotherapy, 3D-CRT, intensity modulated radiotherapy (IMRT), and VMAT [16, 17]. Field abutment is difficult and may result in cold or hot dose spots. The dose of cold spot resulted in low dose at the target, which is one cause of tumor recurrence. A hot spot dose can lead to serious complications, and radiation myelopathy has been reported in the literature [13].

HT is a new revolutionary technology that can complete CSI in one session, and the abutment between irradiation fields is avoided. Compared with 3DCRT and IMRT, HT can provide better dose uniformity and conformity in CSI plan [7, 8]. In this study, data in Table 3 showed that *V*_95%_ of HT, VMAT, and 3D-CRT was close to 100%, and the lowest value is 97.58% of patient 5 with 3D-CRT. The maximum *V*_107%_ with HT was 4.09%, and the remaining values fluctuated between 0 and 0.39%. The minimum *V*_107%_ with VMAT was 0.5%, and the remaining values fluctuated between 2.28% and 20.62%. The two smaller *V*_107%_ values were 3.09% and 3.31% in VMAT, and the remaining values fluctuated between 38.87% and 63.84%. The difference in *V*_107%_ between HT and 3D-CRT reached statistical difference, while that between VMAT and 3D-CRT nearly reached statistical difference. These results indicated that the target volume covered with high dose was minimum with HT, the maximum value with 3D-CRT, and the value of VMAT was between HT and 3D-CRT. *V*_107%_ was obviously different in individuals. The difference could be associated with individual body shape and thickness.

There was overall statistical difference in DHI and CI among HT, VMAT, and 3D-CRT. In this study, data showed that with HT the DHI values were close to 1, and the CI values fluctuated between 0.85 and 0.92, which was similar to that reported in the literature [8, 18]. Dose uniformity and conformity are good for the HT plan, which can provide ideal dose distribution. The DHI values of VMAT and 3D-CRT were also close to 1, and the maximum value was 1.13 with 3D-CRT. It was indicated that the uniformity was also good with VMAT and 3D-CRT. The CI values of VMAT fluctuated between 0.83 and 0.88. The CI values of VMAT of patient 2 and patient 3 were slightly higher than that of HT. It was indicated that the conformity of VMAT was also good. The CI value of 3D-CRT fluctuated between 0.66 and 0.74; therefore the conformity of 3D-CRT was poor.

There was statistical difference in time among HT, VMAT, and 3D-CRT. The beam-on time of HT was the longest, and the time fluctuated between 605 and 682 seconds, which was similar to that reported in the literature [19]. The beam-on time of VMAT which fluctuated between 326 and 330 seconds was relatively long. The beam-on time of 3D-CRT which fluctuated between 66 and 87 seconds was the shortest. Before the application of CSI, image registration also took a relatively long time, but the HT plan could be completed in one setup. Image registration before treatment is conducive to the accurate implementation of the treatment. Conventional radiation technology application requires a number of setups, and treatment technicians need journeys to and from treatment room and control room to complete the radiation field abutment, which is also time-consuming. The beam-on and image registration time of HT treatment is relatively long, while the beam-on time of conventional technique is short. However, the conventional technique needs several times setup, and the application of abutting fields requires a relatively long time. Study showed that the procedures from setup to the completion of CSI required about 45 minutes in HT, and other techniques required similar time [19].

The OAR volume exposed to high doses decreased in the HT plan, while the OAR volume exposed to low doses increased. It was a major drawback of HT [7, 8].  Table 4 showed that *V*_5%_ and *V*_10%_ of OAR were high, reaching 100% in some cases with HT. The dose of OAR dropped rapidly from *V*_20%_, and some rapidly fell to 0, similar to the results reported in the literature [8]. Although the volume of lung exposed to low dose was large, no patients developed symptomatic acute radiation pneumonitis [20]. The effect of large volume exposed to low dose needs to be confirmed in a long-term follow-up.

As showed in Table 5, *V*_5%_ and *V*_10%_ of OAR were also high with VMAT, and dose dropped rapidly. *D*_1%_ of lens was lowest with 3D-CRT. *D*_1%_ of the other OAR was the highest with 3D-CRT. At left lung, right lung, heart, liver, stomach, left kidney, and right kidney dose fall of 3D-CRT was slower than that of VMAT and HT. This is because of fixed radiation beams with 3D-CRT. Irradiation dose was low in organs away from the beam path, while the irradiation dose was high in organs that were in the beam path. Compared with HT and VMAT, there was poor conformity, small dose gradient, and slow dose fall in 3D-CRT. Sharma et al. [8] reported that the exposed dose to lung with 3D-CRT was lower than that with HT, but our result was contrary. This situation may be caused by the three radiation beams that irradiated relatively a lot of volume of lung with our 3D-CRT plan.

Ovaries are sensitive to radiation. Up to 50% of ovarian follicles can be destroyed by 2 Gy irradiation [21]. The ovaries are inevitably irradiated in CSI, and the irradiation can result in infertility caused by ovarian function damage. Ovary transplantation was used before the start of CSI. After the transplantation, the irradiation dose exposed to ovary reduced significantly, and ovarian function could be preserved [22]. There have also been reports of reduced ovarian doses and that ovarian function could be preserved with the use of proton irradiation [23]. Three female patients in this study were treated with CSI, without ovary protection. Menstrual disorders occurred in the 3 female patients during the treatment, and 2 patients whose menstrual volume increased significantly suffered 3rd-degree hemoglobin inhibition. At the time of follow-up, patient 3 had normal menstruation but was not pregnant. Patients 4 and 5 had amenorrhea. In this group, the irradiation dose to which the ovaries of the 3 female patients were exposed could not be evaluated. However, from the dose results of the other OAR, we could infer that a large ovarian tissue volume was exposed to low doses. Ovarian function could be significantly affected by CSI irradiation. To preserve ovarian function, ovary transplantation should be performed before CSI treatment. Ovary protection should be strengthened in the process of CSI planning.

The incidence of AMB is low, and the use of HT in the treatment of AMB has been reported less frequently. This small sample in a retrospective study showed that the application of CSI with HT is convenient for clinical practice, and the side effects are mild. Attention should be paid to the protection of the ovary function in female patients in CSI. For good dose uniformity and conformity, VMAT can also be the treatment selection for CSI. Meanwhile the target volume exposed to high dose (*V*_107%_) with VMAT was higher than that of HT, and HT is more convenient for clinical practice. The uniformity is also good with 3D-CRT, but the conformity is poor. So 3D-CRT should not be the priority selection for CSI. Because of small sample size, incomplete data of the patients, nonuniform radiation dose, and short follow-up time, the long-term effect of HT treatment about AMB should be confirmed by larger sample size and longer follow-up times.

## Figures and Tables

**Table 1:** General characteristics of the patients and the radiotherapy-related parameters.

Category	Patients				
	Patient 1	Patient 2	Patient 3	Patient 4	Patient 5
General characteristics					
Gender	Male	Male	Female	Female	Female
Age (year)	30	25	28	18	23
Tumor location	Cerebellopontine angle area	Fourth cerebral ventricle	Cerebellum	Cerebellar vermis	Cerebellar vermis
Neurological examination	Negative	Negative	-	Negative	Negative
Tumor size (cm)	3.0 × 3.1	2.4 × 3.6	-	3.5 × 4.3	2.8 × 3.0
Histological features	Group 4	Group 4	- ^*∗*^	Group 4	Group 4
Extension of tumor	Cerebellum	Third ventricle of cerebrum	-	Fourth cerebral ventricle	Fourth cerebral ventricle
The amount of surgical removal (cm)	3.0 × 3.0 × 2.0	4.0 × 6.0 × 3.0	-	4.5 × 4.5 × 3.0	3.5 × 3.5 × 3.0
MRI					
Brain MRI/tumor residual	Yes/no	Yes/yes	Yes/no	Yes/no	Yes/no
Spinal cord MRI/metastasis	Yes/no	Yes/yes	No/-	No/-	No/-
Posttreatment MRI/progressive disease	Yes/no	-^#^	Yes/no	Yes/no	Yes/no
Radiotherapy-related parameters					
TM stage	T_2_M_0_	T_4_M_3_	T_X_M_X_	T_2_M_X_	T_3a_M_X_
Prescription dose (Gy/F)					
PTV1	30.6/17	35/20	30.6/17	36/20	36/20
PTV2	19.8/11	18/10	23.4/13	18/10	18/10
Interruption/cause	Yes/ bone suppression	Yes/ vacation, pain	Yes/ machine breakdown	Yes/ bone suppression	No/-
Interruption time (day)	4	5	2	3	0

*∗*: the patient underwent surgery at another hospital in Shanghai City, and the pathology data cannot be obtained, and the histological characteristics of the patient cannot be furtherly determined; #: the patient abandoned treatment after 7 fractions of radiotherapy and had no MRI reexamination. And the patient died in July 2017.

**Table 2:** Treatment toxicity and clinical treatment.

Category	Patients				
	Patient 1	Patient 2	Patient 3^#^	Patient 4	Patient 5
Acute toxicity grade					
Leukocyte	3	1	0	2	3
Neutrophils	2	0	0	1	2
Hemoglobin	0	0	0	3	3
Platelet	3	1	0	2	0
Hair loss	2	0	2	2	2
Clinical treatment	IL-11, G-CSF, Anti-infection, platelet transfusion	Pain management	-	IL-11, G-CSF	G-CSF, EPO

#: no blood samples reexamined after 3 weeks of radiotherapy; G-CSF: recombinant human granulocyte colony stimulating factor; EPO: erythropoietin; IL-11: interleukin-11.

**Table 3:** Target dose parameters.

Category	Patient														
	Patient 1			Patient 2			Patient 3			Patient 4			Patient 5		
	HT	VMAT	3D-CRT	HT	VMAT	3D-CRT	HT	VMAT	3D-CRT	HT	VMAT	3D-CRT	HT	VMAT	3D-CRT
Time	682	326	74	728	326	67	676	333	72	605	333	66	610	330	87
*V*_95%_	99.22	98.81	98.95	99.83	99.02	99.67	97.69	99.92	99.93	99.45	99.49	99.45	99.80	98.65	97.58
*V*_107%_	0	16.00	63.84	4.09	20.62	38.87	0.37	0.50	3.09	0.01	8.47	3.31	0.39	2.28	51.36
*D*_1%_	32.32	33.82	35.02	37.83	39.06	39.12	33.05	32.66	33.12	37.55	38.92	39.14	38.22	38.62	42.07
*D*_99%_	29.38	28.80	29.04	35.42	33.28	34.08	27.14	30.37	30.08	34.84	35.30	34.80	35.51	33.70	30.14
*D*_mean_	31.54	31.92	32.93	36.61	36.71	37.27	32.18	31.71	31.75	36.71	37.59	37.16	36.81	37.29	38.45
DHI	1.05	1.08	1.13	1.04	1.09	1.09	1.08	1.05	1.07	1.03	1.06	1.07	1.04	1.06	1.10
CI	0.87	0.83	0.68	0.85	0.87	0.66	0.86	0.87	0.68	0.85	0.85	0.68	0.92	0.88	0.74

Time: beam-on time (second); *V*_*n*%_: *n*% prescription dose delivered to percent volume; *D*_1%_: irradiation dose delivered to 1% volume, representing the maximum dose; *D*_99%_: irradiation dose delivered to 99% volume, representing the minimum dose; *D*_mean_: average irradiation dose.

**Table 4:** Statistical analysis results of target dose parameters among HT, VMAT, and 3D-CRT.

Parameters	Category						
	Mean (SD)			*P *value			
				Overall *P* value	*P* value among groups		
	HT	VMAT	3D-CRT		HT versus VMAT	HT versus 3D-CRT	VMAT versus 3D-CRT
Time	660.20 (52.17)	329.60 (3.51)	73.20 (8.41)	0.00	0.00	0.00	0.00
*V*_95%_	99.20 (0.88)	99.18 (0.52)	99.12 (0.93)	0.99	0.97	0.87	0.90
*V*_107%_	0.97 (1.75)	9.57 (8.66)	32.09 (27.81)	0.03	0.44	0.01	0.06
*D*_1%_	35.79 (2.86)	36.62 (3.11)	37.69 (3.58)	0.65	0.69	0.37	0.60
*D*_99%_	32.46 (3.92)	32.29 (2.64)	31.63 (2.62)	0.91	0.93	0.68	0.74
*D*_mean_	34.77 (2.67)	35.04 (2.97)	35.51 (2.97)	0.92	0.88	0.69	0.80
DHI	1.05 (0.02)	1.07 (0.02)	1.09 (0.02)	0.02	0.15	0.01	0.09
CI	0.87 (0.03)	0.86 (0.02)	0.69 (0.03)	0.00	0.57	0.00	0.00

Time: beam-on time (second); *V*_*n*%_: *n*% prescription dose delivered to percent volume; *D*_1%_: irradiation dose delivered to 1% volume, representing the maximum dose; *D*_99%_: irradiation dose delivered to 99% volume, representing the minimum dose; *D*_mean_: average irradiation dose.

**Table 5:** Irradiation dose of OAR: mean (SD).

OAR	Category								
	*D*_1% _(Gy)	*D*_mean _(Gy)	*V*_5%_	*V*_10%_	*V*_20%_	*V*_30%_	*V*_40%_	*V*_50%_	*V*_80%_
*Left lens*									
Tomo	4.75 (0.88)	3.67 (0.57)	100 (0)	69.90 (36.35)	0 (0)	0 (0)	0 (0)	0 (0)	**0 (0)**
VMAT	4.72 (0.50)	4.31 (0.50)	100 (0)	100 (0)	0 (0)	0 (0)	0 (0)	0 (0)	**0 (0)**
3D-CRT	3.34 (0.94)	2.92 (0.92)	93.49 (10.66)	34.19 (44.71)	0 (0)	0 (0)	0 (0)	0 (0)	**0 (0)**
Tomo	4.86 (0.97)	3.83 (0.64)	100 (0)	78.17 (34.88)	0 (0)	0 (0)	0 (0)	0 (0)	**0 (0)**
*Right lens*									
VMAT	4.92 (0.59)	4.22 (0.49)	100 (0)	100 (0)	0 (0)	0 (0)	0 (0)	0 (0)	**0 (0)**
3D-CRT	3.79 (1.18)	3.11 (0.99)	99.50 (1.12)	51.10 (47.55)	0 (0)	0 (0)	0 (0)	0 (0)	**0 (0)**
Tomo	23.75 (3.93)	11.60 (2.39)	100 (0)	96.80 (4.47)	75.64 (9.52)	53.21 (9.37)	37.32 (9.72)	22.05 (9.06)	**0.03 (0.08)**
*Left eye ball*									
VMAT	15.19 (2.49)	7.21 (1.30)	100 (0)	99.99 (0.02)	45.10 (18.34)	16.52 (10.00)	4.95 (3.80)	0.65 (0.67)	**0 (0)**
3D-CRT	28.58 (7.32)	7.58 (1.79)	98.42 (2.24)	69.77 (22.76)	31.89 (10.41)	22.63 (9.06)	16.30 (7.55)	11.91 (6.85)	**4.42 (3.74)**
Tomo	24.79 (3.40)	12.09 (2.09)	100 (0)	97.62 (3.37)	73.99 (6.30)	56.33 (8.13)	40.18 (8.49)	25.16 (7.93)	**0.29 (0.58)**
*Right eye ball*									
VMAT	17.72 (3.59)	8.85 (3.26)	100 (0)	100 (0)	50.21 (13.79)	20.26 (6.20)	7.84 (3.77)	1.10 (1.56)	**0 (0)**
3D-CRT	33.66 (3.30)	10.17 (2.87)	98.54 (1.13)	75.66 (17.78)	41.62 (12.74)	32.40 (10.80)	26.53 (9.87)	21.22 (10.22)	**11.67 (6.31)**
Tomo	11.09 (2.55)	4.09 (0.72)	98.06 (4.35)	56.32 (15.37)	8.63 (4.63)	1.96 (1.92)	0.51 (0.63)	0.20 (0.19)	**0.01 (0.01)**
*Left lung*									
VMAT	12.10 (2.20)	3.80 (4.59)	92.88 (3.70)	45.74 (5.01)	9.05 (2.74)	2.10 (1.00)	0.64 (0.50)	0.21 (0.21)	**0 (0.01)**
3D-CRT	14.56 (2.53)	4.42 (0.90)	72.37 (12.45)	42.73 (11.34)	26.87 (10.26)	8.91 (5.09)	1.73 (1.64)	0.44 (0.49)	**0.02 (0.04)**
Tomo	13.41 (3.20)	4.31 (0.83)	98.25 (3.81)	56.84 (14.69)	12.01 (6.10)	3.63 (3.03)	1.31 (1.35)	0.48 (0.54)	**0.01 (0.01)**
*Right lung*									
VMAT	14.46 (2.72)	4.08 (0.71)	91.63 (7.84)	49.67 (8.58)	11.22 (4.20)	3.62 (1.57)	1.34 (0.80)	0.51 (0.42)	**0.02 (0.03)**
3D-CRT	17.18 (3.56)	5.27 (1.18)	77.95 (12.97)	48.95 (11.38)	33.89 (9.40)	14.93 (6.10)	4.77 (3.65)	1.62 (1.83)	**0.03 (0.08)**
Tomo	11.18 (2.64)	5.45 (0.90)	100 (0)	91.07 (8.18)	21.44 (15.88)	3.95 (6.94)	1.62 (2.38)	0.24 (0.54)	**0 (0)**
*Heart*									
VMAT	14.44 (6.53)	6.85 (3.0)	100 (0)	86.22 (12.99)	38.56 (33.63)	16.88 (26.68)	8.15 (16.49)	4.19 (9.36)	**0.28 (0.64)**
3D-CRT	17.03 (3.64)	5.40 (1.07)	73.36 (8.98)	47.40 (11.08)	36.08 (10.64)	21.59 (13.29)	8.07 (6.87)	0.67 (1.04)	**0 (0)**
Tomo	9.24 (1.66)	3.97 (0.91)	95.78 (9.33)	58.35 (21.37)	7.07 (4.04)	0.66 (0.76)	0.05 (0.10)	0 (0.01)	**0 (0)**
*Liver*									
VMAT	12.75 (2.68)	4.42 (0.95)	87.83 (9.23)	53.87 (13.51)	18.38 (10.56)	4.96 (4.09)	1.22 (1.23)	0.47 (0.70)	**0 (0)**
3D-CRT	17.07 (2.78)	5.01 (1.08)	69.19 (13.03)	45.24 (11.78)	31.54 (9.79)	16.95 (7.54)	4.89 (2.73)	0.97 (0.68)	**0 (0)**
Tomo	8.16 (1.62)	4.96 (0.53)	100 (0)	93.96 (5.04)	9.22 (7.29)	0.09 (0.14)	0 (0)	0 (0)	**0 (0)**
*Stomach*									
VMAT	14.60 (4.55)	7.19 (2.56)	96.95 (6.80)	85.28 (19.45)	53.20 (31.90)	18.75 (19.52)	4.88 (5.70)	1.20 (1.83)	**0 (0)**
3D-CRT	17.54 (5.30)	6.43 (2.62)	81.30 (17.83)	57.95 (21.38)	44.47 (17.91)	20.90 (14.30)	7.06 (6.97)	4.10 (5.51)	**0 (0)**
Tomo	11.25 (2.39)	6.52 (1.30)	100 (0.01)	99.28 (1.60)	39.70 (23.27)	4.91 (4.20)	0.21 (0.31)	0 (0)	**0 (0)**
*Left kidney*									
VMAT	7.62 (4.82)	3.61 (2.09)	92.09 (9.99)	36.37 (36.44)	9.26 (19.08)	1.81 (3.49)	0.24 (0.46)	0 (0)	**0 (0)**
3D-CRT	14.45 (6.42)	3.33 (1.27)	70.35 (16.60)	19.05 (14.56)	12.44 (13.79)	9.02 (11.44)	3.49 (4.87)	1.51 (2.14)	**0 (0)**
Tomo	9.43 (1.46)	6.15 (0.78)	100 (0)	99.82 (0.40)	29.92 (9.91)	0.61 (1.08)	0 (0)	0 (0)	**0 (0)**
*Right kidney*									
VMAT	5.93 (2.22)	3.50 (1.75)	92.61 (9.53)	39.47 (35.46)	6.17 (13.55)	0 (0)	0 (0)	0 (0)	**0 (0)**
3D-CRT	13.48 (3.78)	4.21 (0.56)	78.08 (17.11)	35.52 (8.48)	25.11 (8.49)	10.41 (7.29)	1.34 (2.24)	0.34 (0.76)	**0 (0)**

*V*_*n*%_: *n*% prescription dose delivered to percent volume.
